# Evaluation of precipitated CaCO_3_ produced from locally available limestone as a reinforcement filler for PVC pipe

**DOI:** 10.1038/s41598-024-58594-7

**Published:** 2024-05-16

**Authors:** Addis Lemessa Jembere, Melkamu Birlie Genet, Bantelay Sintayehu

**Affiliations:** Faculty of Chemical and Food Engineering, Bahir Dar Institute of Technology, Bahir Dar University, Bahir Dar, Ethiopia

**Keywords:** PVC, Filler, CaCO_3_, Compounding, Chemical engineering, Nanoscale materials

## Abstract

The current experimental work aimed at developing PCC through two major process steps: dissolution and precipitation, using raw materials domestically available as SL, which are intensively used in construction inputs. The pH level was the decisive parameter used to determine the time required to complete the dissolution and carbonation processes during precipitation. The optimal pH levels were found to be 13 for dissolution and 7.1 for precipitation, respectively. The produced PCC was characterized based on chemical analysis, crystallinity, and morphology, showing an increment of CaCO_3_ content exceeding 99%, sharper crystal peaks, and predominantly calcite PCC. The compatibility of the PCC was assessed by incorporating 25%, 50%, 75%, and 100% of PCC with commercial filler, followed by selected mechanical tests, such as stress at yield, density, and elongation at break. The results indicated that mixing ratios of 25%, 50%, and 75% of PCC with the commercial filler met the standards, with stress at a yield above 45 MPa and density within the range of 1.35 to 1.46 g/cm^3^. However, complete substitution slightly lowered these properties. Nevertheless, the elongation at break was acceptable at all treatment levels.

## Introduction

Limestone is a predominant type of sedimentary rock in the Earth's crust, primarily composed of CaCO_3_^1^. Calcium carbonate ranks among the most abundant naturally occurring minerals, comprising over 5% of the Earth's crust. Limestone is predominantly found in Ethiopia, notably within the Jurassic Antalo limestone in the central part of the country and the Hamanlei Series in the east-central region. It occurs abundantly in various parts of Ethiopia, with significant deposits in Somali, Oromia, Amhara, and Tigray national regional states^2^. The most noteworthy exposures and deposits of Antalo Limestone are in the central Abay Valley, as well as side valleys like Jema, Wonchit, and Muger valleys^3^. Sizeable limestone deposits also exist in the eastern part of Ethiopia, particularly in the Harar-Hakimgara areas^4^.

However, the required qualities of CaCO_3_ minerals vary across industries that utilize this mineral as a filler and feedstock. To produce high-quality natural CaCO_3_, the rock's purity, whiteness, and homogeneity must be suitable, and even then, extensive treatment may be necessary^5^. CaCO_3_ plays a crucial role in various industries, with the construction industry being the largest consumer, using it as a building material. Additionally, the iron industry employs CaCO_3_ for purification, while the oil industry incorporates it into drilling fluids. Limestone is relatively soft and can be easily ground into a fine, non-toxic, usually white powder, making it an ideal filler in numerous products where cost-effective bulk addition is essential.

Calcium carbonate stands as the most widely used filler^6^ and extender material in industries such as paper^7^, paint, plastic, sealants, adhesives, food, ceramics, textiles (carpet), cosmetics, medicine, and several others^8^. Different industries have specific requirements for product characteristics, including chemical purity, particle size distribution, shape, surface area, whiteness, and rheological behavior. There are two primary sources of calcium carbonate worldwide: GCC and PCC^9^. GCC is extracted from the Earth and exists in various forms like calcite, aragonite, vaterite, limestone, chalk, marble, or travertine. After extraction, GCC is ground under either dry or wet conditions, depending on the desired final product. On the other hand, PCC can crystallize in three main polymorphs: calcite (rhombohedral), aragonite (orthorhombic), and vaterite (hexagonal), influenced by reaction conditions and impurities in the process^10^. Calcite is the most thermodynamically stable under ambient conditions, but other polymorphs can form under specific kinetic conditions. Aragonite, denser and more soluble than calcite, typically forms needle-like orthorhombic crystals, favored at high temperatures and pressures but slowly converting to calcite. Vaterite, the least stable polymorph, forms hexagonal crystals and is rarely found in nature^11^. Both GCC and PCC can be used as a PVC filler but PCC exhibits finer particle size, possesses a regularity of shape, Narrowness of particle size distribution, and high purity, which makes it a better candidate for the application as a reinforcement filler. Furthermore, Incorporating GCC as a filler reduces raw material costs although, it has a high higher specific gravity, which is 2.5–3 times higher than the requirement on a volumetric basis^12^.

Most pipes used for irrigation and sewerage systems are made from Polyvinyl chloride (PVC). However, virgin PVC exhibits mechanical instability. To improve its mechanical properties, fillers are added. Virgin PVC tends to be brittle, requiring additives to enhance its impact resistance^13^. Fillers are also incorporated into PVC resin mixes to reduce material costs, provide coloration, offer ultraviolet (UV) protection, and assist in lubrication. Generally, calcium carbonate (limestone) serves as a common filler used to replace resin.

With the increased construction activity in Ethiopia, the plastic industry has expanded significantly. In this thriving sector, mineral fillers are fundamental components for producing durable, high-quality products. Among these mineral fillers, calcium carbonate minerals are predominant. Plastic industries import calcium carbonate mineral fillers, and the demand for these fillers is rising. Consequently, producing calcium carbonate filler from locally available limestone presents a sustainable solution to address these challenges.

Commercial precipitated calcium carbonate (PCC) production dates back to 1841 G.C., when it was first produced by the English company John E. Sturge Ltd. They treated residual CaCl2 from their KClO_3_ production unit with Na_2_CO_3_ and CO_2_ to create PCC^1^. In 1898, the first milk of lime process was implemented in Birmingham^14^. Numerous studies have explored PCC production from various sources, including recovering calcium hydroxide for PCC production from SL in automobile welder's carbide sludge^15^. Other studies have investigated high-purity calcium carbonate recovery via a pH-swing process using hydrochloric acid, ammonium hydroxide, and carbon dioxide, examining key process parameters such as acid amount (HCl/calcium molar ratio), pH, and CO_2_ flow rate^16^. Additionally, researchers have studied calcium carbonate precipitation from hydrated lime with variable reactivity, granulation, and optical properties^17^. A study also highlighted PCC production from power plant fly ashes^18^. Another area of investigation explored the possibility of reducing CO_2_ emissions by producing calcium and magnesium carbonates from silicate materials for long-term CO_2_ storage via multi-step processes^19^. Authors have investigated mineral carbonation using carbon dioxide gas^20,21^ as well as dissolution properties of steelmaking slags in acetic acid for PCC production^22^. Furthermore, studies have examined the effect of limestone characteristics and calcination temperature on lime quality^23^.

This research primarily focuses on the feasibility of substituting imported calcium carbonate fillers with locally available materials for PVC pipe applications. The study investigates the impact of adding locally produced PCC at varying mixing ratios with commercial fillers and assesses compatibility in terms of mechanical properties. This research is pioneering in its exploration of the potential of locally available limestone to replace the in-demand commercial filler. It serves as an initial step toward further research into applicability, quality enhancement, and commercialization. The optimal reaction time is determined through subsequent pH measurements, and the effect of PCC on commercial fillers is examined with regard to mechanical properties such as stress at yield, elongation at break, and density.

## Materials and methods

### Dissolution–precipitation (DP)

**Figure 1:**
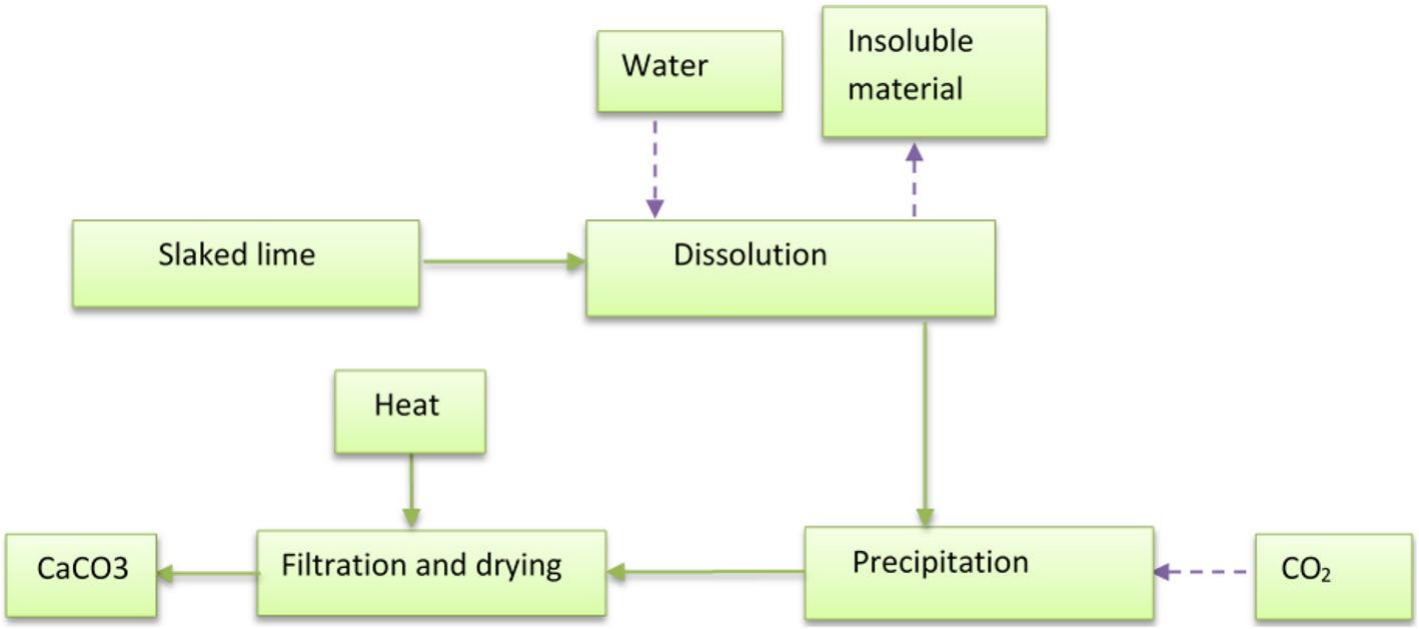
Experimental process flow diagram.

In this experiment, the carbonation method was selected to produce PCC by following the experimental process diagram shown in Fig. 1. Pre-calcined SL was obtained from the Derba cement factory, along with 99.9% pure CO_2_ from the Dashn brewery factory in Gonder, Ethiopia, as the primary feedstock for PCC production. Subsequently, SL was dissolved in distilled water to initiate the dissolution process, leading to the formation of PCC, as outlined in Eq. (1).

1 $${\text{CaO }}\left( {\text{Calcined lime}} \right) \, + {\text{ H}}_{{2}} {\text{O}} \to {\text{Ca }}\left( {{\text{OH}}} \right)_{{2}} \left( {\text{Hydrated lime}} \right) \, + {\text{ Heat}}$$

In the dissolution step, the SL to distilled water was set at 1:4 (w/v). Precipitation reaction was conducted under atmospheric pressure (1 bar) and at a room temperature of 25 °C. In this process, the Ca(OH)_2_ solution was subjected to a reaction with CO_2_, and an excess of CO_2_ was introduced into the reactor to ensure complete conversion. The extent of the precipitation reaction was easily monitored by continuously measuring the solution's pH during the precipitation tests. The initial pH of the Ca(OH)_2_ solution before the reaction was 13. The pH of the precipitation reaction was measured at 10-min intervals for up to 1 h. Following precipitation, the resulting PCC was filtered and subsequently dried in an oven at a temperature of 120 °C for 24 h.

To facilitate the dissolution process, the SL solution was stirred at a constant temperature of 25 °C and a stirring speed of 150 rpm, varying the mixing time. We measured the pH of the SL solution at 5-min intervals, ranging from 5 to 35 min. Subsequently, we determined the optimal dissolution time when the pH stabilized. The subsequent dissolution step was carried out at this optimized time. After completing the dissolution process, the non-dissolving fraction was removed by sieving through a 100 μm sieve. The pure calcium hydroxide solution was then subjected to a reaction with CO_2_ to produce PCC through a precipitation reaction, as depicted in Eq. (2). The reaction between the Ca(OH)_2_ solution and CO_2_ gas took place in the Autoclave.

2 $${\text{Ca }}\left( {{\text{OH}}} \right)_{{2}} \left( {\text{Hydrated lime}} \right) + {\text{ CO}}_{{2}} \leftrightarrow {\text{ CaCO}}_{{3}} \left( {{\text{PCC}}} \right) \, + {\text{ H}}_{{2}} {\text{O}}$$

### Characterization

Characterization of both SL and PCC with regard to their crystalline structure was carried out using an X-ray Diffraction (XRD), Minflux 300/600 powder XRD Rigaco (USA). The apparatus was configured with a Cu tube and a CuKα graphic monochromator radiation source, featuring a wavelength (*λ*) of 1.540593 Å and a scanning speed of 10°/min. The scan ranged from 10 to 70° in 2-Theta, with a step size of 0.02. The recorded results were presented in terms of 2-Theta (degrees) versus intensity (count). For examining the morphology of both SL and PCC fillers, scanning electron microscopy (SEM) was employed as a valuable tool. The morphological characteristics of the samples were analyzed using the FE-SEM/FIB-model Neon-40 (Field emission scanning electron microscope) at the Nanomanufacturing Technology Center (NMTC), CMTI. The particle size distribution was also calculated using the ImageJ version 1.54d^24^ by measuring as many pixels as possible. The normality particle size distribution was determined statistically using the Shapiro–Wilk test using RStudio version 2022.07.2 + 576^25^. The chemical oxide compositions of SL and PCC were characterized through a silicate analysis, which included analytical LiBO2 Fusion, HF attack, Gravimetric, Colorimetric, and Atomic absorption spectroscopy (TL-1800AA)^26–29^. This oxide compositional analysis was conducted at the Ethiopian Geological Survey in Addis Ababa.

### PVC compounding

**Figure 2:**
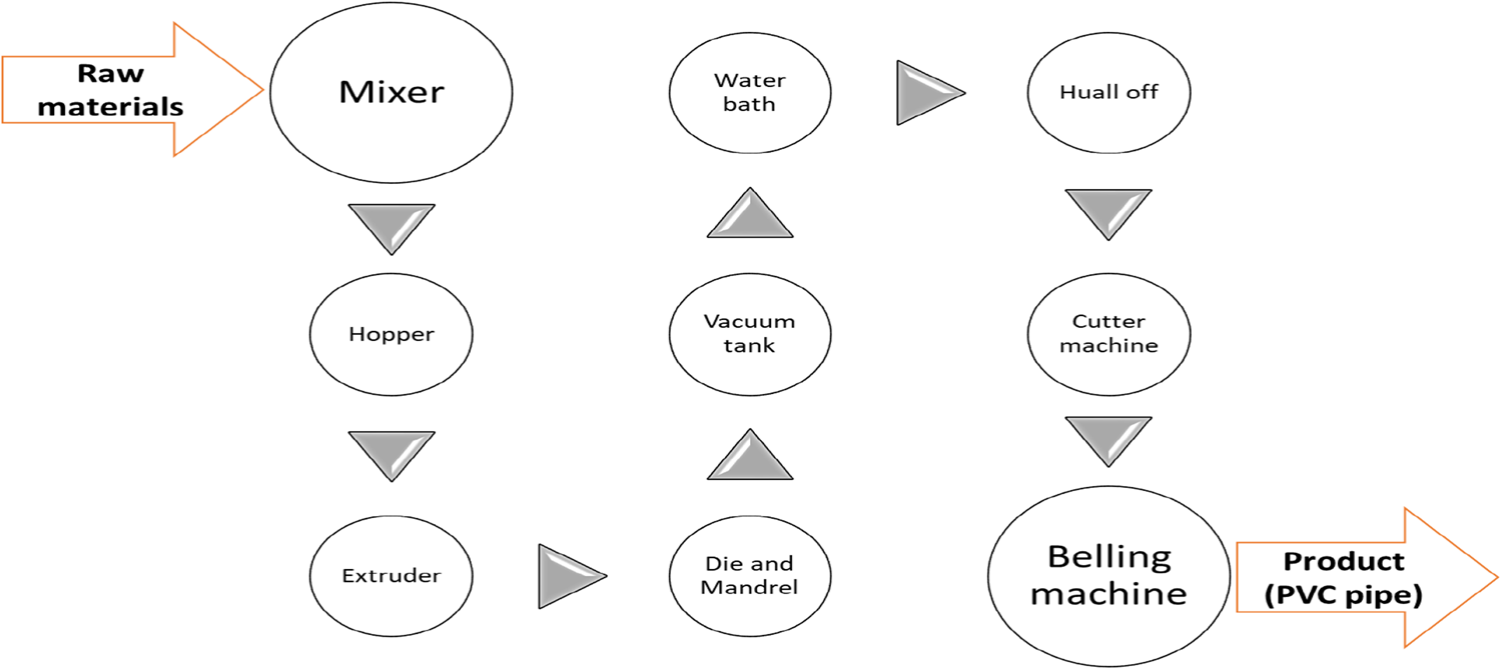
PVC processing process flow diagram.

The formulation for manufacturing PVC pipes with a D_out_ of 160 mm and a PN of 10 MPa is detailed in Table 1. This compounding process was carried out at the Amhara pipe factory according to the generalized block flow diagram shown in Fig. 2. To produce a single batch of 160 mm diameter PVC pipes, varying amounts of PCC, ranging from 25 to 100% by weight were combined with a commercial filler. The choice of material handling procedures for creating the primary mix formulations was influenced by the characteristics of the PVC resins and other additives used in PVC compounds. This research primarily employed the prevalent processing method for PVC compound formulation, known as dry blending or powder mixing, in accordance with ASTM D2396^30^. Dry blending, the industry standard, involves initially processing over 85% of suspension and bulk PVC resins by blending each PVC compounding additive in batch mode using a standard mixer. The formulation included adding 15 PHR of calcium carbonate filler, as indicated in Table 1. The process involved mixing PVC resin powder, stabilizer powder, calcium carbonate powder, titanium dioxide powder, and solid carbon black in a mixer. A high-temperature mixer, operating at approximately 120 °C, combined these materials at high speed. Once the required temperature was reached, the mixer automatically discharged the mixture into a cooling chamber, reducing the temperature to around 50 °C. Subsequently, pneumatic transportation was used to transfer the mixed materials to a hopper for moisture content removal, followed by transfer to an extruder for melting and achieving homogeneity. The material was then molded using die machines, with the internal diameter adjusted by a Mandrel and the external diameter controlled by the die. The formed pipes were cooled in a vacuum tank equipped with circulating water spray for efficient cooling. Additional cooling was achieved by submerging the pipes in a water bath tank, accompanied by spraying, which was essential to prevent shape deformation resulting from sudden cooling. The hull-off machine provides a constant pulling rate to maintain the proper wall thickness of the finished product followed by cutting according to the required length and finally fitting of the socket at the end of the pipe by using Belling machine.

**Table 1:** PVC compounding ingredients in PHR.

Material and additives	Amount
PVC	100
CaCO_3_*	15
Stabilizer	4
Titanium oxide	0.1
Carbon black	0.0025

*PCC to commercial filler ratio varied from 25 to 100% by weight.

### Mechanical properties of PCC-reinforced PVC

Mechanical properties involved tensile stress at yield, elongation at break, and density measurements. Tensile testing, as per ESISO-6259^31^, involves subjecting a sample to controlled tension until it reaches failure. Parameters directly measured through this test include ultimate tensile strength (stress at yield) and maximum elongation. Specific gravity, which is a measure of density relative to a reference substance (pure water in this case), was determined following ESISO 1183^32^ guidelines. If a material has a specific gravity of less than 1, it will float on water. This test method entails weighing a single-piece specimen measured between 1 to 50 g in water, using a sinker made of materials lighter than water. It is suitable for plastics that can become wet but are otherwise unaffected by water. The test specimen consists of a single piece of the material being tested, with no specific size or shape restrictions as long as its volume remains under 1 cm^3^, and its surface and edges are smoothed. The thickness of the specimen for the specific gravity test was adjusted to 1 mm having 1 g of weight.

## Result and discussion

### Effect of pH on the dissolution of the slaked lime and PCC

**Figure 3:**
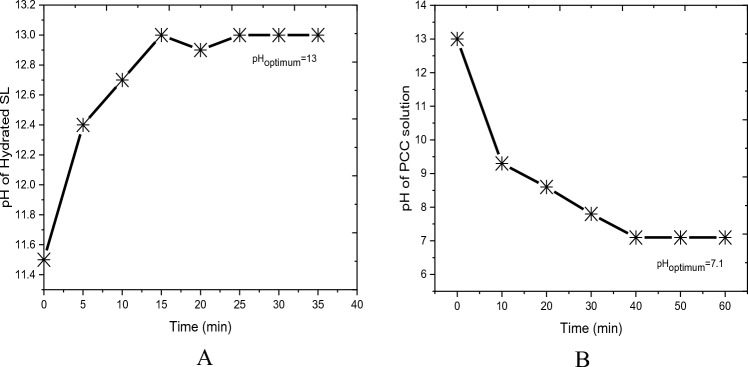
Effect of precipitation time on the (**A**) pH of hydrated SL and (**B**) PCC solution.

The dissolution of 1 kg slaked lime sample in 4L water i.e. 1:4 w/v ratios at 25 °C was investigated by varying the dissolution time from 0 to 35 min with increments of 5-min intervals at the fixed mixing rate of 150 rpm. As can be depicted in Fig. 3, the pH of the dissolved solution was varied with the dissolution time of slaked lime. The pH increased with an increase of dissolution time up to 15 min but remained steady at 13 pH value with further increases of dissolution times. The results depicted that 15 min was enough (optimum) to dissolve the slaked lime powder sample. Therefore, the subsequent precipitation experiments were carried out at this optimum dissolution time (15 min).

The precipitation reaction of the dissolved slaked lime with CO_2_ gas in the autoclave was investigated at 1 bar constant pressure and 25 °C temperature for different precipitation times of 10, 20, 30, 40, 50, and 60 min. Figure 3 shows that the pH of the solution decreased as the precipitation time increased. The pH of the solution decreased markedly from 13 to pH 8.6 in 20 min of precipitation after which, the pH decreased slowly until the 40th min, which recorded a pH of ~ 7. The slow decrease in pH was explained by the formation of carbonic acid in the autoclave during precipitation and hence 40 min was selected as the optimum precipitation time for further synthesis and characterization.

### Compositional analysis

The chemical analysis of the SL powder sample is presented in Table 2. The slaked lime powder mainly contains 62.95% CaO and a higher percentage was LOI (27%). The minor oxides presented in small amounts are SiO_2_ (4.12%), Al_2_O_3_ (1.16%), and MgO (1.05%). SL also contains minor constituents less than 1% i.e. Fe_2_O_3_ (0.5%), Na_2_O (0.39%), and K_2_O (0.13%). Chemical analysis of the synthesized PCC sample is also presented in Table 2. Chemical analysis of the produced PCC has increased substantially as a result of precipitation reaction to a purity of 99.2% of CaCO_3._ The purity of PCC used for PVC pipe formulation should have a purity greater than 98% CaCO_3_ that complies with the standard.

**Table 2:** Chemical composition of SL and PCC samples.

Chemical constituents (%)	SL	PCC-1
CaO	62.95	
CaCO_3_	–	99.2
SiO_2_	4.12	0.07
Al_2_O_3_	1.16	0.09
Fe_2_O_3_	0.5	0.001
MgO	1.05	0.15
SO_3_	1.46	0.12
K_2_O	0.13	–
Na_2_O	0.39	–
LOI	27.67	0.36

### Thermal analysis

**Figure 4:**
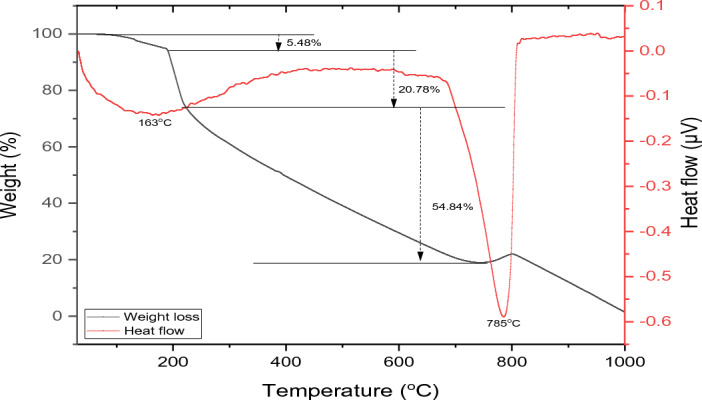
DSC-TGA of synthesized CaCO_3_.

TGA was conducted to assess the thermal stability of the samples at different temperatures. Dynamic thermogravimetric and DTG curves are shown in Fig. 4.

The thermogravimetric analysis curve illustrated the weight change of the sample during thermal decomposition over the temperature range of 40–1000 °C. The thermal properties of the PCC are characterized by three main weight loss steps, from 108 to 190 °C, 190 °C to 228 °C, and from 228 to 1000 °C. Corresponding to a 5.48, 20.78 and 72.18 wt. %, respectively. The first phase is attributed to the evaporation of physisorbed water, which are weakly bonded water molecules^33,34^ and decarboxylation might occur in the first phase. The second weight loss is attributed to the removal of strongly physisorbed water and was attributed to dehydration and crystallization of amorphous CaCO_3_.^35^. The larger weight loss observed was the third phase, which was attributed to the loss of CO_2_ from the carbonate decomposition. This high percentage of this phase also provided an independent confirmation that the samples were comprised almost solely of CaCO_3_ phases. The DSC curve shows two endothermic phases. The two phases were associated with a broad endothermic peak at 163 °C and a sharp endothermic peak recorded at high temperature (~ 785 °C) which is associated with the melting point of the sample decomposing into CaO_2_.

### Crystallinity analysis

**Figure 5:**
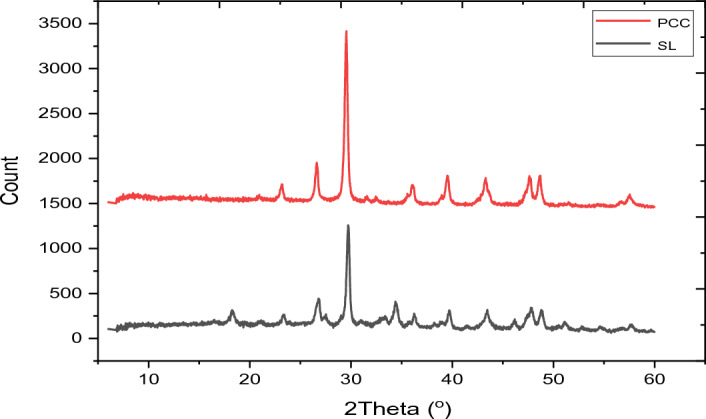
XRD of SL and synthesized PCC sample.

The XRD patterns of PCC demonstrate that there is a slight shift in peak position but the difference is mostly related to the intensity and the sharpness of XRD profiles at the same peak positions. The XRD pattern is presented in Fig. 5. The analysis revealed a difference in structure between raw SL and PCC because the treatment method induce variability in the peak positions and modulation of their intensities. Synthesized PCC has two major intense diffraction peaks at 2θ of 26.58°and 29.54°, which represent Calcite and aragonite, respectively. These peaks appear similarly in SL with values of 26.54° and 29.75° but the intensity is lower than the PCC indicating the formation of concentrated CaCO_3_ because of dissolution and precipitation process. PCC also possesses smaller peaks at 2θ of 23.14° which represent the Aragonite type and 2θ at 36.02°, 39.6°, 43.24°, 47.52°, 48.62° and 57.62° in calcite form. Furthermore, the peak at 18.16° in the case of SL was absent in PCC showing the possible development of a crystalline phase. The residual peaks of Ca(OH)_2_ noted might be due to the incomplete calcination process.

### Surface morphology

**Figure 6:**
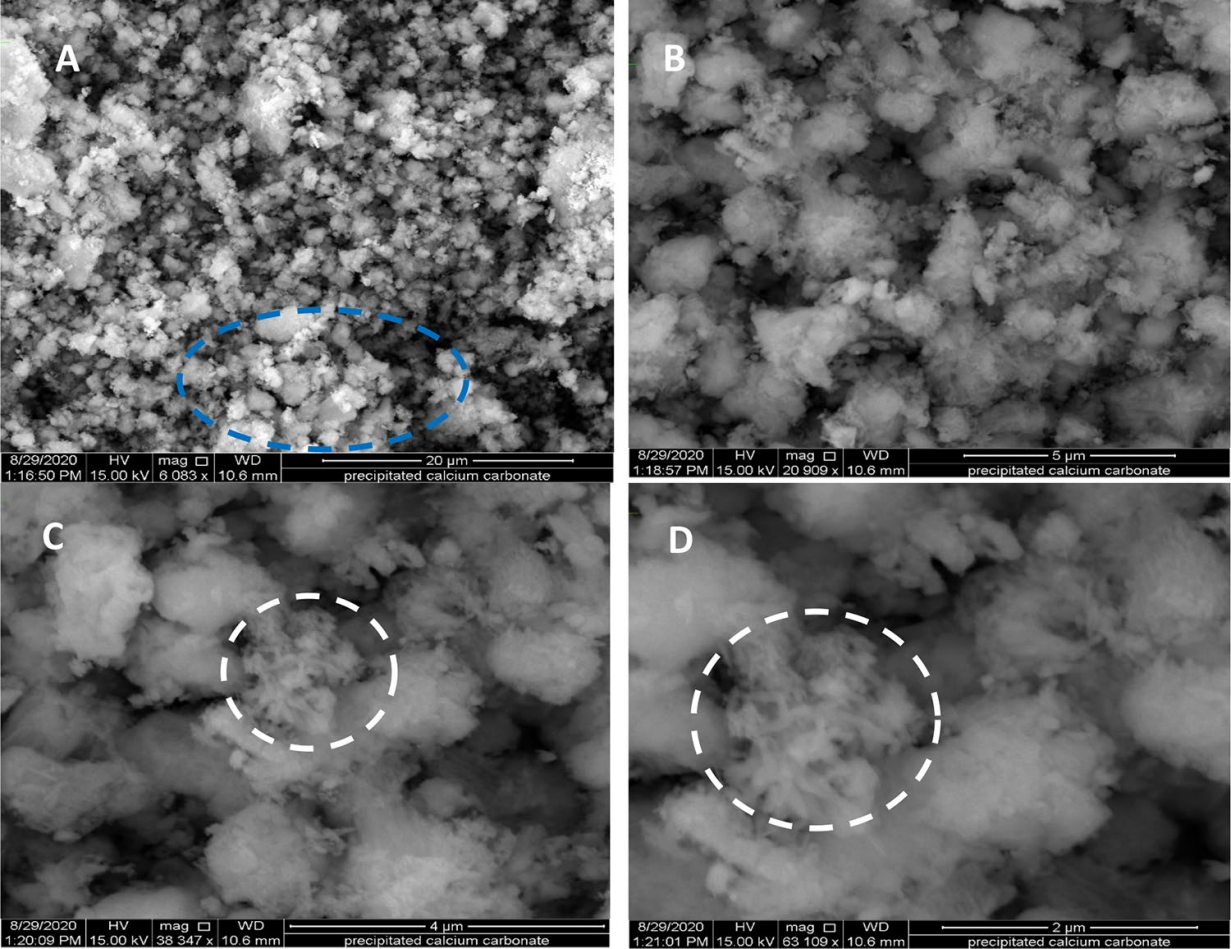
SEM image for PCC nanoparticle at different magnifications (**A**) 20 μm (**B**) 5 μm (**C**) 4 μm and (**D**) 2 μm.

**Figure 7:**
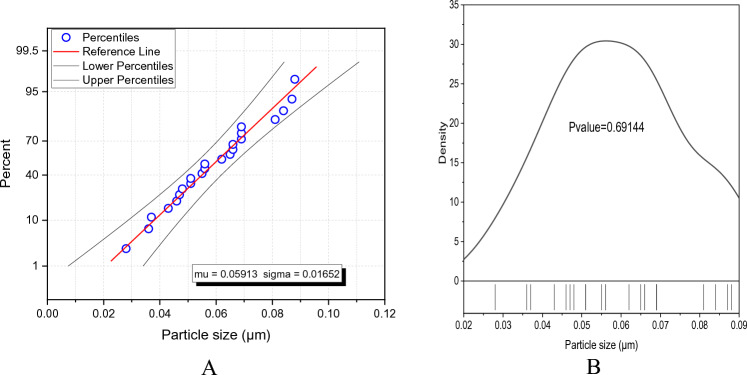
(**A**) Probability plot and (**B**) Density curve for PCC particle size.

The surface morphology of the PCC particles was studied by SEM images as depicted in Fig. 6A–D at different magnifications. Micrographs obtained under different magnifications revealed aggregated mineral flocs (blue circle in Fig. 6A) in the PCC. The images show the existence of both calcite and aragonite (Fig. 6B) crystal structure that possesses different crystal growth patterns. The rod-shaped aragonite crystals are arranged in bundles in a semi-circular structure (white circle in Fig. 6C, D). Similar micrograph observation was reported by previous research^36^. PCC sample appears to be heterogeneous in terms of particle size and shape. It exhibits a narrow size range and rhombohedral crystal shape. The mean particle size of the PCC is found to be 0.05913 μm, which is near the median value of 0.056 indicating the normal distribution of the particle size. The size range of the representative particles is between 0.028 and 0.088 µm. Furthermore, the Shapiro–Wilk test for normality test shows a *P*_value_ much higher than *α* (0.05) indicating that the representative particles measured come from normally distributed particles and the normality curve shown in Fig. 7B resembles a Gaussian plot. Figure 7A also indicates the measured particle size follows the Weibull distribution.

### Performance analysis of reinforced PVC pipe

**Figure 8:**
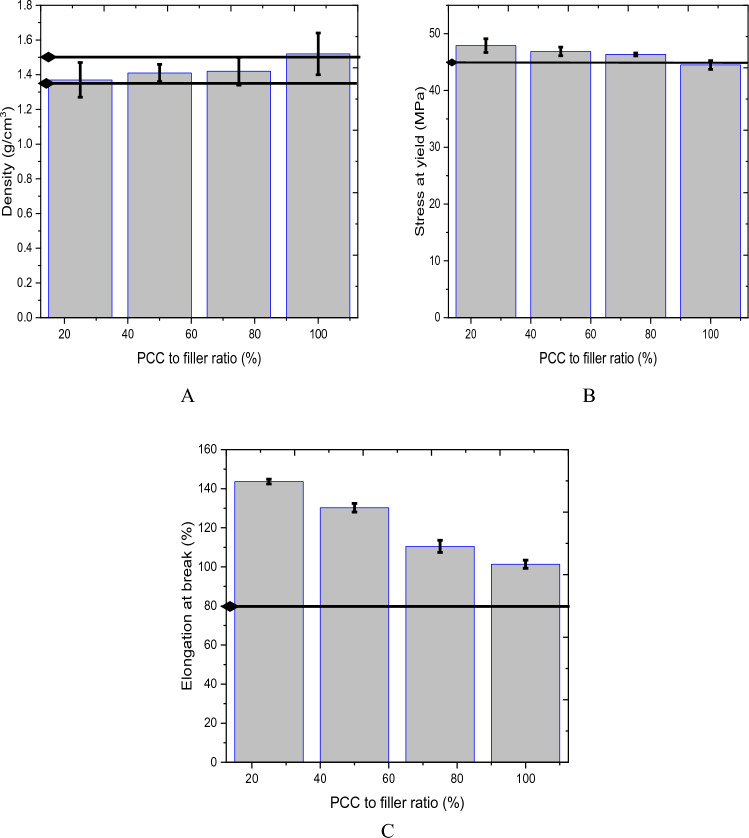
Effect of filler ratio on (**A**) density, (**B**) Stress at yield, and (**C**) elongation at break.

Figure 8 shows the filler-mixing ratio against Stress at yield, elongation at break, and density. Overall, the corresponding density values prepared from different filler ratios are within close agreement with each other except for the complete substitution of commercial filler with PCC. As seen from Fig. 8A, the PCC to commercial filler ratio from 25 to 75% resulted in a density within the standard (1.35 to 1.46 g/cm^3^) recording 1.37 to 1.42 g/cm^3^ respectively and 100% substitution resulted in higher density of 1.52 g/cm^3^ than the standard. The decrease in density of the composite pipe is attributed to using finer PCC filler indicating that the commercial filler has a smaller particle size than PCC. The use of high-strength fillers is often cost-prohibitive. Here the purity and the particle size of PCC filler significantly affect the mechanical property of the PVC pipe.

Figure 8B represents the effect of varying PCC filler ratios on the stress at yield of the developed PVC composites. PCC filler ratio with the commercial filler at 25%, 50%, and 75% is within the standard (> 45 MPa) resulting in 47.9, 46.87, and 46.35, respectively. Complete replacement of PCC still results in a slight reduction (44.5 MPa) from the standard as shown by the reference (black line) from Fig. 8C. The decrease in tensile strength at 100% utilization of PCC causes increased brittleness, and this can be attributed to the formation of agglomerates in PCC. These agglomerates form initiation spots of stress concentrations that lead to failure^37,38^. Figure 8C depicted that the PVC sample filled with the filler at all ratios resulted in an elongation at a break above the standard (80%). However, increasing the PCC ratio decreased the elongation at break from 143.64% to 101.35%. This test shows how the commercial filler is more ductile before it breaks. While the synthesized PCC is stiffer, the brittleness increases when increasing the ratio.

## Conclusion

Based on the results, the synthesized PCC exhibits significant potential as a filler for compounding PVC, replacing a substantial portion of commercial filler. The pH level emerged as a critical parameter for both dissolution and precipitation processes, with optimal pH values determined to be 13 for dissolved SL and 7.1 for carbonation. Analysis of the synthesized PCC revealed that it primarily consisted of Calcite, with the presence of Aragonite as well, a finding consistent with observations from SEM images. Further examination of chemical analysis indicated that the treated PCC exhibited higher purity (99.2%), aligning with the standard requirements for fillers in the PVC industry. Incorporating PCC alongside commercial CaCO_3_ at varying mixing ratios yielded mechanical strength values within acceptable industry limits for stress at yield and density when the PCC content ranged from 25 to 75%. However, complete replacement led to a slight reduction in these properties. Notably, the elongation at break for all mixing ratios conformed to established standards. This research underscores the potential of locally available CaCO_3_ as a promising raw material for filler applications. The preliminary experiment signals the possibility of replacing commercial silica with this locally sourced material.

## Data Availability

The datasets used and/or analyzed during the current study available from the corresponding author on reasonable request.

## List of symbols

PCC: Precipitated calcium carbonate
PVC: Polyviniyle chloride
SL: Slaked lime
GCC: Ground calcium carbonate
XRD: X-Ray diffraction
D_out_: Outer diameter
PHR: Parts per Hundred Rubber
TGA: Thermogravimetric analysis
DTG: Derivative Thermogravimetry
PN: Pressure nominal
